# Immunoreactivity of Polish Lyme Disease Patient Sera to Specific *Borrelia* Antigens—Part 1

**DOI:** 10.3390/diagnostics11112157

**Published:** 2021-11-21

**Authors:** Iwona Wojciechowska-Koszko, Magdalena Mnichowska-Polanowska, Paweł Kwiatkowski, Paulina Roszkowska, Monika Sienkiewicz, Barbara Dołęgowska

**Affiliations:** 1 Department of Diagnostic Immunology, Immunology and Laboratory Medicine, Pomeranian Medical University in Szczecin, Powstancow Wielkopolskich 72, 70-111 Szczecin, Poland; pawel.kwiatkowski@pum.edu.pl (P.K.); paulina.roszkowska@pum.edu.pl (P.R.); 2 Department of Medical Microbiology, Immunology and Laboratory Medicine, Pomeranian Medical University in Szczecin, Powstancow Wielkopolskich 72, 70-111 Szczecin, Poland; magdalena.polanowska@pum.edu.pl; 3 Department of Pharmaceutical Microbiology and Microbiological Diagnostic, Medical University of Lodz, Muszynskiego St. 1, 90-151 Lodz, Poland; monika.sienkiewicz@umed.lodz.pl; 4 Department of Laboratory Medicine, Immunology and Laboratory Medicine, Pomeranian Medical University in Szczecin, Powstancow Wielkopolskich 72, 70-111 Szczecin, Poland; barbara.dolegowska@pum.edu.pl

**Keywords:** Lyme disease, immunoreactivity, ELISA, immunoblot, *Borrelia*

## Abstract

The diverse clinical picture and the non-specificity of symptoms in Lyme disease (LD) require the implementation of effective diagnostics, which should take into account the heterogeneity of *Borrelia* antigens. According to available guidelines, laboratories should use a two-tier serological diagnosis based on the enzyme-linked immunosorbent (ELISA) screening test and confirmation of the immunoblot (IB). The aim of the study was to investigate the immunoreactivity of LD patient sera to *Borrelia* antigens and to attempt to identify the genospecies responsible for LD using an ELISA–IB assay combination. Eighty patients with suspected LD and 22 healthy people participated in the study. All samples were tested with ELISA and IB assays in both IgM and IgG antibodies. In the case of the ELISA assay, more positive results were obtained in the IgM class than in the IgG class. In the case of the IB assay, positive results dominated in the IgG class. Positive results obtained in the IB assay most often showed IgM antibodies against the OspC and flagellin antigens, whereas the IgG antibodies were against VlsE, BmpA, OspC, p41, and p83 antigens. The IB assay is an important part of LD serodiagnosis and should be mandatory in diagnostic laboratories.

## 1. Introduction

Lyme borreliosis (LB) or Lyme disease (LD) is a multi-organ disorder caused by tick-borne spirochaetes of the *Borrelia burgdorferi* sensu lato species complex. It has been a reportable communicable disease in the European Union since 2014. Three clinically relevant species—*B. afzelii*, *B. garinii*, and *B. burgdorferi* sensu stricto—transmitted by *Ixodes ricinus*, are mostly responsible for the disease in Europe and also in Asia. *B. garinii* and *B. afzelii* are predominant species spread all over Europe. *B. burgdorferi* sensu stricto is distributed mainly in Western European countries [1]. An increasing number of infections, caused by other *Borrelia* species such as *B. spielmanii*, *B. bavariensis*, and *B. lusitaniae*, have also been reported [2,3,4,5]. LD is mostly characterized in the first stage by erythema migrans (EM)—a red skin rash or lesion that spreads in rings from the site of the bite, which appears in about 60–90% of patients about 1 week after a tick bite and can last up to 4 weeks, after which it disappears spontaneously. In rare cases, erythema chronicum migrans—a characteristic inflammatory skin lesion—may appear, or other severe manifestations involving the patient’s skin, nervous system, joints, or heart [6,7]. It is worth emphasizing that, according to literature data on cases of neuroborreliosis in children, the incidence of EM was estimated to be about 30% in European countries. This information requires increased vigilance while diagnosing LD [8]. In addition, it was found that cases of LD in Europe may be characterized by non-specific clinical symptoms mimicking other neurological diseases [9]. Diverse clinical signs and non-specific symptomatology of LD can lead to misdiagnosis, delayed recognition, or overdiagnosis [10]. Microbial or serological confirmation of LD is needed for all clinical courses of the disease except for pathognomonic EM [2,11]. Serological detection of *Borrelia*-specific antibodies is still the laboratory method of choice in LD diagnosis [2,3,11,12,13]. According to the Centers for Disease Control and Prevention (CDC) recommendations, the serological standard practice requires a two-tier testing algorithm, otherwise ambiguous results can complicate the diagnosis. The first-tier assay is enzyme-linked immunosorbent assay (ELISA) or indirect immunofluorescence assay (IIFA) [14,15]. All reactive results, reported with screening tests, must be retested with a separate IgM and IgG immunoblot (IB) confirmatory test (second-tier assay) on the same serum sample [11,14,15,16].

The choice and processing of antigens (markers) for IB is still a great challenge because of borrelial antigen heterogeneity; therefore, kits/tests are continuously being improved [3,17]. Nowadays, recombinant anti-*Borrelia* IB kits are recommended because conventional ones using whole-cell antigens can generate non-specific immunoreactivity, show limited agreement between each other, and require complicated interpretation. The result achieved with the use of recombinant IB is easier to standardize and to establish a diagnosis. The use of species-specific recombinant antigens improves blot specificity and can identify the genospecies responsible for LD [18,19,20,21,22].

The aim of the study was to investigate the immunoreactivity of Polish Lyme disease patient sera to genospecies-specific *Borrelia* antigens and to attempt to identify genospecies responsible for LD using an ELISA—IB assay combination.

## 2. Materials and Methods

### 2.1. The Study Group

Eighty individuals from the West Pomeranian region (Poland) who had developed LD in Europe and 22 healthy individuals (control group) were included in the study. All the patients were diagnosed on the basis of clinical symptoms and serological findings. The following symptoms of LD were observed: single EM (*n* = 57), borrelial lymphocytoma (*n* = 3), Lyme arthritis (*n* = 17), and peripheral neuropathy (*n* = 3). Ten LD patients required hospitalization in the Clinical Hospital in Szczecin, Poland (Clinic of Skin and Venereal Diseases, *n* = 8; Clinic of Internal Diseases, *n* = 1; and Clinic of Pediatrics, Hematology, and Oncology, *n* = 1). Each LD patient reported exposure to a tick-bite and after 25 ± 2 days following the exposure, a serological diagnosis was conducted. None of the LD patients were treated with anti-*Borrelia* antibiotics before serum collection for serological testing. Healthy blood donors were used as negative-control sera (*n* = 22). None of the controls reported a tick bite or demonstrated LD symptoms at the time of examination. All serum samples were negative for a rheumatoid factor, screened by ELISA and retested by IB.

### 2.2. ELISA Assay

Sera samples were tested by ELISA kit (EUROIMMUN, Lübeck, Germany) in the IgM and IgG antibody class. All assays were performed in the same laboratory using a procedure suggested in the manufacturer’s protocols. IgG was identified on the basis of whole-cell lysate antigens of *B. burgdorferi* sensu stricto, *B. afzelii*, and *B. garinii*, as well as the VlsE recombinant protein of *B. burgdorferi*. IgM was detected with whole-cell lysate antigens of *B. burgdorferi* sensu stricto, *B. afzelii*, and *B. garinii*. Results of the quantitative analysis of both immunoglobulin types were expressed in RU/mL (relative units/mL). Sera ≥22 RU/mL were considered positive and those <16 RU/mL were considered negative. Results ranging from 16 to 22 RU/mL were ambiguous (“borderline”) according to European recommendations [23]. Manufacturer-suggested cut-off levels as well as an interpretation of results were applied.

### 2.3. Immunoblot Assay

Patients’ sera reactivity was verified by means of the anti-*Borrelia* confirmatory assay anti-*Borrelia* EUROLINE-RN-AT (IgM and IgG; EUROIMMUN, Lübeck, Germany) regardless of the result of ELISA screening. All tests were performed in the same laboratory using the procedure suggested in the manufacturer’s protocols. The following target antigens were used to detect antibodies of the IgG class: recombinant VlsE of *B. afzelii*, *B. garinii*, and *B. burgdorferi*; recombinant and highly specific antigens of *B. burgdorferi*: p21, p20, p19, p18, recombinant flagellin (p41), and BmpA (p39) of *Borrelia*; recombinant and dimeric OspC; recombinant protein of *Borrelia:* p83; and lipid of *B. afzelii* and *B. burgdorferi*. Antibodies of the IgM class were identified with recombinant VlsE of *B. burgdorferi*, recombinant flagellin (p41), and BmpA (p39) of *Borrelia*, as well as recombinant and dimeric OspC advanced antigens of *B. afzelii*, *B. garinii*, *B. burgdorferi*, and *B. spielmanii*. IB results were analyzed using EUROLineScan software (EUROIMMUN, Lübeck, Germany) in accordance with the interpretive criteria of the manufacturer [24].

### 2.4. Statistical Analysis

The statistical analysis of the results was conducted using a test for independence of χ^2^ or a test for independence of χ^2^ with Yates’ correction on Statistica 6.0 Pl software (StatSoft, Inc, Palo Alto, CA, USA). Probability *p* of the first kind of error (the level of test significance), equal to 0.05, was considered acceptable.

## 3. Results

Among the sera samples of 80 LD patients, only 60 (75.0%) and 45 (56.2%) were found to be ELISA-positive for IgM and IgG classes, respectively. The remaining samples were seronegative when tested by the ELISA assay. IB verified both ELISA-positive and ELISA-negative samples in both types of antibodies. IgM IB revealed 39 positive samples, 34 of which appeared to be ELISA-positive and 5 appeared to be ELISA-negative. In the IgG class, there were 53 blot-positive sera, 38 of which were ELISA-positive and 15 ELISA-negative. It is noteworthy that we observed anti-*Borrelia* blot reactivity in ELISA-negative samples that did not require a confirmatory assay in routine serodiagnostics or with IB.

These false-negative ELISA samples were found in both types of antibodies. The difference in the number of positive IgM samples was statistically significant (*p* < 0.0006) when compared to ELISA and IB. The percentage of positive IgM and IgG blots following a negative ELISA was statistically insignificant. An analysis of only one type of antibody confirmed that the ELISA test revealed IgM-positive sera (26%), whereas IB was associated with more IgG-reactive (26%) sera. A comparable number of samples appeared to be seroreactive in both classes of antibodies tested with the application of ELISA or IB. In the control group of 22 subjects, two ELISA-reactive sera (9.1%) were observed for each class, but only one was confirmed with IB. The number of IgM and IgG seropositive samples revealed with ELISA and IB for the study and control groups is presented in Table 1.

In the group of 57 patients with EM, 39 (68%) had anti-*Borrelia* blot-IgM. None of the 20 patients with late stages of infection (Lyme arthritis, peripheral neuropathy) presented anti-*Borrelia* IgM.

The most frequent IgM-blot reactivity of false-negative ELISA samples was against OspC—adv of *B. afzelli* (5 patients) and flagellin (p41, 4 patients) (Table 2).

**Figure 1:**
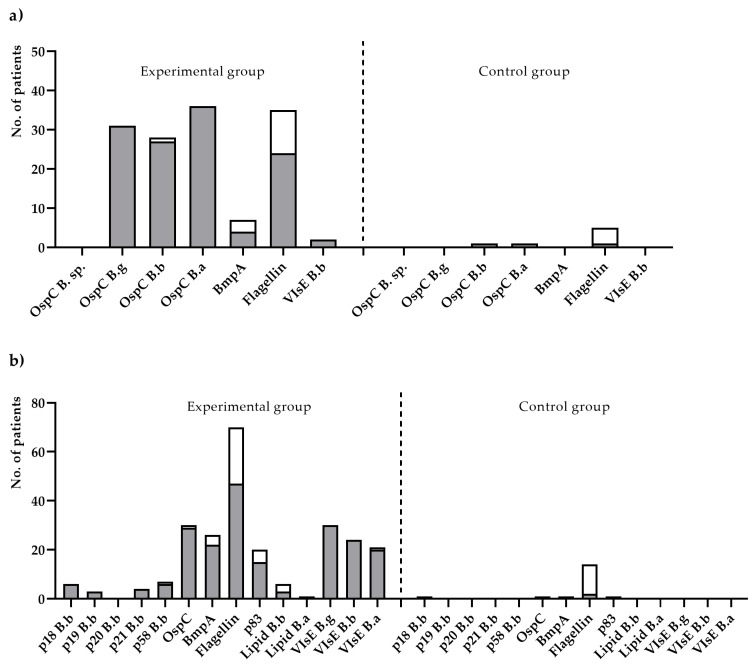
The number of positive results obtained from Lyme disease patients (experimental group) and healthy individuals (control group) for the IgM (**a**) and IgG (**b**) antibody types for particular antigens, included in the immunoblot assay. The white-colored mark is the number of antigens obtained from positive results; the grey-colored mark is the number of antigens obtained from negative results.

IgM-blot reactivity of all 39 LD sera was observed mostly against species-specific OspC variant antigens and flagellin (p41) of *B. afzelii* (24; 61.5%). A reaction with OspC in a blot test for IgM was found for *B. afzelli*, *B. garinii*, and *B. burgdorferi* in 36 (92.3%), 31 (79.5%), and 27 (69.2%) LD sera, respectively. As for IgM antibodies against other *Borrelia*-antigens, BmpA of *B. afzelii* and VlsE were rarely detected: in four (10.2%) and two (5.1%) LD sera, respectively. IgG-blot reactivity was detected for multiple antigens of three *Borrelia* genospecies: p41, VlsE variants (*n* = 35; 66%), OspC, and BmpA of *B. garinii* and p83. As for IgG against lipids of *B. burgdorferi* and *B. afzelii* as well as against characteristic antigens of *B. burgdorferi*, BB_A34, BB_P38, BB_K53, and BB_N38 were rarely found (Figure 1).

IgM and IgG seroreactivities to *Borrelia g*enospecies in LD are shown in Table 3. Seroreactivity against markers of single genospecies of *Borrelia* sensu lato was occasionally detected in both classes. IgM-blot antibodies reacting with antigens of *B. afzelli* were most frequently found (10.3%)*,* whereas predominant IgG-blot antibodies were detected against OspC, flagellin, and VlsE of *B. garinii* antigens (13.2%). The blot antibodies against VlsE of *B. afzelli* antigens were found only in the IgG type (1.9%).

Usually, seroreactivity against two or three *Borrelia* genospecies was found. IgM seroreactivity against two genospecies (*B. afzelii* and *B. burgdorferi* (4 sera)) and against *B. afzelii* and *B. garinii* antigens (6 sera) was noted in 25.6% of patients. IgG seroreactivity against two genospecies (*B. burgdorferi* and *B. garinii* (7 sera)) and *B. garinii* and *B. burgdorferi* (3 sera) was noted in 18.9% of patients. IgG usually recognized flagellin, BmpA, specific recombinant VlsE blot antigen of *B. garinii*, and p83 *B. burgdorferi*. IgM and IgG antibodies against various antigen combinations of three *Borrelia* genotypes were observed in 24 (61.5%) and 15 (28.3%) patients, respectively. The most frequently detected IgM against three genotypes reacted to species-specific OspC, flagellin and BmpA of *B. afzelii*, and VlsE of *B. burgdorferi*, whereas IgG reacted to the following antigens: species-specific VlsE, flagellin and BmpA of *B. garinii*, OspC of *B. garinii*, and p83 of *B. burgdorferi*.

A detailed summary of ELISA and IB results for anti-*Borrelia* IgM and IgG antibodies in LD patients (experimental group) and in healthy individuals (control group) is shown in Supplementary Materials.

## 4. Discussion

A two-step algorithm is recommended worldwide in diagnosis of LD because of the divergent sensitivity and specificity of both tests [2,6,17]. Serological parameters of the ELISA–IB assay combination can support a clinical suspicion of LD or contradict it [4,7]. The current study with the application of *Borrelia* IB confirmed that IgM-positive ELISA results are significant, thereby improving the specificity of serodiagnosis and showing high sensitivity of ELISA to the IgM class. In the study, ELISA positive results and negative IgM blots for patients with EM did not exclude diagnosis of LD. Thus, from the point of view of Ang et al. [17], high sensitivity of ELISA could be helpful to diagnose early stages of LD with clinical manifestation when the blot is still negative. A selection of samples that need confirmatory blotting is also proposed [17].

The percentage of IgG-positive blots following a negative ELISA was insignificant but indicated that the use of ELISA as a standalone assay in the detection of LD developed in Poland can result in false-negative results for 25% and 43% of samples in IgM and IgG classes, respectively. An analysis of false-negative ELISA results was carried out by Ang et al. [17], who found that 36% (4/11) of the ELISA-negative samples after using EUROIMMUN IB appeared to be blot positive. Our current study is consistent with findings that suggest the use of IB in LD-suspected patients with negative ELISA results [16]. In the case of an analysis of our results, a good reference is a publication by Tracy and Baumgarth [25]. They demonstrated in a mouse model that *B. burgdorferi* completely disrupt the architecture of the germinal centers of lymphoid tissues, disrupting the normal process of “class switch” from IgM to IgG and resulting in persisting IgM reactivity. In our work, all patients were “early,” which might explain some of the cases’ failure to evolve a positive screening test since this can sometimes require the passage of time. Then again, some patients never develop a positive ELISA yet have fully diagnostic WB of either IgM or IgG or both long into the illness and sometimes a very expanded pattern of IgM bands without IgG bands. It suggests that these are not false, but represent ongoing antigen-processing by the immune system of antigens presented by a chronic infection.

In this study, IgM-blot-positive sera against OspC variants of three genospecies and non-specific flagellin (p41) were most frequently found, as was confirmed by Wilske et al. [2], whereas IgG-blot-positive sera reacted to multiple antigens of three genospecies: VlsE antigen variants, p41, OspC, BmpA (p39), and p83. OspC (outer surface protein C) is an immunodominant protein of the IgM response and is expressed in early stages of LD infection. VlsE (variable surface recombinant antigen E) is recognized early by IgG and is characteristic of late/chronic stages. Many authors believe that antibodies against species-specific OspC and species-specific VlsE are the most reliable serological markers for the detection of *Borrelia* infection and its specificity. A lot of species-specific VlsE variants, primarily expressed in vivo after *Borrelia* infection, can actually be easily detected with a commercially available IgG blot with recombinant VlsE [26,27,28,29,30,31].

The study results showed that the IB kit with species-specific OspC and VlsE markers improved the species specificity of LD serodiagnostics because it was able to identify single *Borrelia* genospecies, which are probably responsible for LD infection. However, the seroreactivity against specific markers of single genospecies was infrequently detected and varied due to the class of antibodies. The IgM-blot-positive sera (*n* = 4) reacted to *B. afzelii* and IgG-positive sera (*n* = 7) reacted to *B. garinii* specific antigens; that is why single genospecies of *B. afzelli* or *B. garinii* could be considered a cause of LD. According to Hubálek and Halouzka [32], *B. afzelii* and *B. garinii* are most frequently detected in Europe; the former is more related to skin lesions and the latter to neuroborreliosis [33]. In the current study, IgM antibodies react to specific antigens of *B. afzelli*, as was observed in four of 57 patients manifesting EM. For *B. garinii*, specific IgG sera were associated with early and late clinical manifestations of LD. The prevalence of *Borrelia* genospecies differs and the results of the study showed some conflicting data with data published by other authors [4,34,35]. The studies of other authors identified mostly anti-*Borrelia* IgM of *B. garinii* and anti-*Borrelia* IgG of *B. afzelii* and *B.* *burgdorferi*. The discrepancies are probably related to different spirochoetal infectivity in ticks, modified by the efficiency of vectors and host factors [34].

It is also worth noting that the seroreactivity against two or three *Borrelia* genospecies was commonly found in both IgM and IgG classes at a level of 87.0% and 73.0%, respectively. Some conflicting data revealed a genetic analysis of *Borrelia* genospecies in ticks. Strube et al. [36] showed that the majority of the ticks (>70%) were infected with one genospecies (20.7% *B. afzelii*, 20% *B. garinii*, 10.4% *B. valaisiana*, 6% *B. spielmanii*, 4.4% *B. burgdorferi*), 28% had two genospecies in various combinations (*B. garinii/B. spielmanii*, *B. garinii/B. valaisiana*, *B. afzelii/B. garinii*, *B. afzelii/B. spielmanii*), and only 1.4% of the ticks carried three genospecies in two different combinations (*B. afzelii/B.* *burgdorferi/B. spielmanii* and *B. garinii/B. spielmanii/B. valaisiana*). According to a study by Rauter and Hartung [37], *B. afzelii* and *B. garinii* are the most common *Borrelia* species, but the distribution of genospecies seems to vary in different regions in Europe. The most frequent coinfection by the *Borrelia* species was confirmed for *B. garinii* and *B. valaisiana*.

The IgM-positive sera usually reacted to OspC and p41 of *B. afzelii* and the IgG-reactive sera recognized mostly flagellin, OspC, and BmpA of *B. garinii* and species-specific VlsE variants. The question is whether immune response towards antigens of multiple borrelial genospecies is a result of co-infection or cross-reactions between antibodies and target antigens used in the IB. So far, the answer to the question is equivocal. The hypothesis of co-infection seems plausible because of the predominant prevalence of IgM/IgG antibodies for multiple *Borrelia* genospecies in the majority of patients recruited to the study. Unfortunately, cross-reactivity between antibodies and target antigens can be neither confirmed nor excluded due to the potentially homologous species-specific OspC and VlsE antigens used in the study. It is still a challenge for manufacturers to improve commercial anti-*Borrelia* tests including highly heterogeneous epitopes characteristic of specific *Borrelia* genospecies. An individualized approach to diagnostics of LD as well as true knowledge of commercial tests and their applications and limitations are required to diagnose LD. Bearing in mind knowledge of LD epidemiology, researchers should use new, standardized commercial tests, such as an ELISA–IB assay combination, which is highly sensitive and specific and enables seroreactivity to genospecies-specific *Borrelia* antigens to be investigated.

It is also worth paying attention to the type of antigens included in the IB tests. The presence of additional antigens in IB tests, as indicated by Liu et al., may increase their specificity (according to their results, the p31 antigen OspA *B. burgdorferi* sensu lato is noteworthy) [1]. Liu et al. applied a modified Western-blot assay, which contained the following recombinant antigens for IgM and IgG class antibodies: p18, p28, p30, p34, p45, p58, p66, p31, p23 (OspC), p41, p93, C6, and VlsE. In addition, these authors also used p39EU and P39US antigens from European and American *B. burgdorferi* sensu lato species, respectively. In our study, conducted with the use of the anti-*Borrelia* EUROLI-NE-RN-AT assay from EUROIMMUN, the recombinant antigens for IgM-class antibodies included VlsE B.b, p41 (B.a), p39 (BmpA B.a), and OspC of three genospecies (B.a, B.b, B.g), and for IgG class antibodies VlsE of three genospecies (B.b, B.a, B.g), p83 B.a, p41 B.g, p39 (BmpA B.g), p25 (OspC B.g), p58 (BB_34), p21(BB_K53), p20 (BB_Q03), p19 (BB_N38), and p18 (BB_P38) were used [24]. The EUROLI-NE-RN-AT assay does not contain the antigens p28, p30, p45, p66, p31 (OspA *B. burgdorferi* sensu lato), p93, or C6. Nevertheless, it contains additional recombinant antigens (e.g., p19, p20, p25) and lipids (B.a and B.b). While choosing a diagnostic test, it is important to ensure it contains recombinant antigens as recommended by the German Society for Hygiene and Microbiology, the Robert Koch Institute, and the CDC (USA) [24].

Apart from to the above-mentioned serological methods for diagnosing LD, other methods are also available. However, they are not used in routine diagnostics. The following methods/assays are available: the Lyme urine antigen test (LUAT) [38]; mass spectrometry, which allows serum LD antigens to be detected [39], the EliSpot C6 Lyme assay for the detection of circulating immune complexes [40]; the recombinant VlsE-based liaison chemiluminescence immunoassay [41]; and the Optiplex *Borrelia* assay, which is interesting and useful and was used in our subsequent research.

## 5. Conclusions

The IB assay should be an indispensable, obligatory element of the two-tier diagnosis of LD, regardless of the results of screening tests. In the current study, we proved that the IB assay did not allow for unambiguous determination of infection specificity in terms of isolating the genus responsible for LD in individual patients despite using key antigens (OspC and VlsE) belonging to three different *Borrelia* genera. These results could suggest that patients are co-infected with two or three *Borrelia* genospecies. However, cross-reactions cannot be excluded in these cases, which may occur because of high homology in the structure of the OspC and VlsE proteins used in the test.

## Figures and Tables

**Table 1:** Comparison of seropositive test results from enzyme-linked immunosorbent (ELISA) and immunoblot (IB) assays for IgM and/or IgG in Lyme disease patients (experimental group) and in healthy individuals (control group).

Serological Test	Result	Experimental Group *n* = 80			Control Group *n* = 22		
		IgM *n* (%)	IgG *n* (%)	IgM and IgG *n* (%)	IgM *n* (%)	IgG *n* (%)	IgM and IgG *n* (%)
ELISA	Positive/borderline	26 (32.5)	11 (13.7)	34 (42.5)	2 (9.1)	2 (9.1)	0 (0.0)
IB	Positive/borderline	12 (15.0)	26 (32.5)	27 (33.7)	1 (4.5)	1 (4.5)	0 (0.0)

**Table 2:** Characteristics of antigens of blot reactivity with false-negative enzyme-linked immunosorbent (ELISA) assay in Lyme disease patients.

Class of Antibody/No. of Patients	Patient No.	ELISA	Immunoblot
**IgM** ***n* = 5**	No. 24	(-)	p41; OspC-adv B.a; OspC-adv B.b
	No. 40	(-)	p41; OspC-adv B.a
	No. 44	(-)	p39 B.a; OspC-adv B.a; OspC-adv B.b
	No. 51	(-)	p41; OspC-adv B.a
	No. 68	(-)	p41; OspC-adv B.a
**IgG** ***n* = 15**	No. 9	(-)	VlsE B.g; p41; p25 (OspC); p21
	No. 10	(-)	p41; p25 (OspC)
	No.25	(-)	p83 B.a; p41; p18
	No. 30	(-)	VlsE B.a; p41; p58; p21; p18
	No. 36	(-)	p83; p41
	No. 37	(-)	p41; p39; p25 (OspC)
	No. 43	(-)	p41; p39; p25 (OspC); p19
	No. 51	(-)	p41; p39; p25 (OspC)
	No. 53	(-)	p41; p39; p25 (OspC)
	No. 56	(-)	VlsE B.b; p41
	No. 60	(-)	VlsE B.b; p41
	No. 62	(-)	p21
	No. 65	(-)	VlsE B.g; p83; p39
	No. 75	(-)	p83; p41; p39; p25 (OspC)
	No. 79	(-)	Vls E B.g; VlsE B.a; p41; p39; p58

**Table 3:** IgM and IgG seroreactivities to *Borrelia g*enospecies in Lyme disease patients (experimental group) and in healthy individuals (control group).

Number of Genospecies	Genospecies	Experimental Group		Control Group	
		IgM *n* (%)	IgG *n* (%)	IgM *n* (%)	IgG *n* (%)
1	*B.* *burgdorferi*	0 (0.0)	6 (11.3)	0 (0.0)	1 (50.0)
	*B. garinii*	3 (7.6)	7 (13.2)	0 (0.0)	0 (0.0)
	*B. afzelii*	4 (10.3)	1 (1.9)	0 (0.0)	0 (0.0)
2	*B.* *burgdorferi* *B. garinii*	0 (0.0)	7 (13.2)	0 (0.0)	0 (0.0)
	*B.* *burgdorferi* *B. afzelii*	4 (10.3)	1 (1.9)	1 (100)	0 (0.0)
	*B. garinii* *B. afzelii*	4 (10.3)	3 (5.7)	0 (0.0)	0 (0.0)
3	*B. burgdorferi* *B. garinii* *B. afzelii*	24 (61.5)	15 (28.3)	0 (0.0)	0 (0.0)
Antigens common to all genospecies		0 (0.0)	13 (24.5)	0 (0.0)	1 (50.0)
Total		39 (100)	53 (100)	1 (100)	2 (100)

## Data Availability

Data are contained within the article.

## Supplementary Materials

The following are available online at https://www.mdpi.com/article/10.3390/diagnostics11112157/s1, Table S1: Detailed summary of enzyme-linked immunosorbent (ELISA) and immunoblot (IB) assay results for anti-*Borrelia* IgM and IgG antibodies in sera samples of Lyme disease patients. Table S2: Detailed summary of enzyme-linked immunosorbent (ELISA) and immunoblot (IB) assay results for anti-*Borrelia* IgM and IgG antibodies in sera samples of healthy individuals (control group).
