# Supplementary material for: Immunoreactivity of Polish Lyme Disease Patient Sera to Specific Borrelia Antigens—Part 1
Source: Diagnostics (Basel). 2021 Nov 21;11(11):2157. doi: 10.3390/diagnostics11112157 (PMC8625222; doi:10.3390/diagnostics11112157)
Supplement: Supplementary file 1 [file diagnostics-11-02157-s001.zip › diagnostics-1453322-supplementary.pdf]

Table S1. Detailed summary of enzyme-linked immunosorbent (ELISA) and immunoblot (IB) assays results for anti-*Borrelia* IgM and IgG antibodies in sera samples of Lyme disease patients.

**No. of patient**	**Days after tick exposure**	**ELISA**		**IB - IgM**							**Result**	**IB - IgG**														**Result**
		**IgM**	**IgG**	VlsE B.b	Flagellin	BmpA	OspC B.a	OspC B.b	OspC B.g	OspC B.sp		VlsE B.a	VlsE B.b	VlsE B.g	Lipid B.a	Lipid B.b	p83	Flagellin	BmpA	OspC	p58 B.b	p21 B.b	p20 B.b	p19 B.b	p18 B.b	
1.	24	**63.484**	**41.513**	-	**(+)**	-	-	-	-	-	NEG.	-	-	-	-	-	**(+)**	**+**	-	-	-	-	-	-	-	NEG.
2.	25	**24.851**	3.706	-	-	-	-	-	-	-	NEG.	-	-	-	-	-	-	**+**	-	-	-	-	-	-	-	NEG.
3.	22	**137.836**	12.655	-	**(+)**	-	**+**	**+**	**(+)**	-	POS.	-	-	-	-	-	-	**+**	-	-	-	-	-	-	-	NEG.
4.	23	**30.027**	**176.807**	-	**(+)**	-	-	-	-	-	NEG	**+**	**+**	**+**	-	-	**+**	**+**	**+**	**+**	**+**	-	-	-	-	POS.
5.	26	4.912	**33.867**	-	-	-	-	-	-	-	NEG.	-	-	-	-	**(+)**	-	-	-	-	-	-	-	-	-	NEG.
6.	27	**71.101**	1.510	-	-	-	**+**	-	-	-	BOR	-	-	-	-	-	-	-	-	-	-	-	-	-	-	NEG.
7.	24	**40.473**	1.388	-	-	-	-	-	-	-	NEG.	-	-	-	-	-	-	-	-	-	-	-	-	-	-	NEG.
8.	26	**52.921**	**102.267**	-	-	-	-	-	-	-	NEG.	**+**	**+**	**+**	-	-	-	**(+)**	-	-	-	-	-	-	-	POS.
9.	24	**(19.117)**	14.960	-	**(+)**	**(+)**	-	-	-	-	NEG.	-	-	**+**	-	-	-	**+**	-	**+**	-	**(+)**	-	-	-	POS.
10.	23	**>200**	11.180	-	**(+)**	-	**+**	**+**	**+**	-	POS.	-	-	-	-	-	-	**(+)**	-	**+**	-	-	-	-	-	BOR.
11.	25	4.209	5.600	-	-	-	-	-	-	-	NEG	-	-	-	-	-	-	**+**	**(+)**	-	-	-	-	-	-	NEG.
12.	22	10.724	**44.112**	-	-	-	-	-	-	-	NEG.	-	**+**	**(+)**	-	-	**(+)**	**+**	-	**(+)**	**(+)**	-	-	-	-	POS.
13.	21	**146.696**	1.692	-	-	-	**+**	**+**	**+**	-	POS.	-	-	-	-	-	-	**+**	-	-	-	-	-	-	-	NEG.
14.	25	7.332	14.738	-	-	-	-	-	-	-	NEG.	-	-	-	-	-	**(+)**	**+**	**(+)**	-	-	-	-	-	-	NEG.
15.	25	**69.018**	**20.512**	-	-	-	**(+)**	**(+)**	-	-	BOR.	-	-	-	-	-	-	**+**	-	**+**	-	-	-	-	-	BOR.
16.	26	12.937	**32.537**	-	-	-	-	-	-	-	NEG.	**+**	**+**	**(+)**	-	-	-	**+**	-	-	-	-	-	-	-	POS.
17.	27	**>200**	**>200**	**+**	**+**	**+**	**+**	**+**	**+**	-	POS.	**+**	**+**	**+**	-	**(+)**	**+**	**+**	**+**	**+**	**-**	-	-	-	-	POS.
18.	28	0.379	0.769	-	-	-	-	-	-	-	NEG.	-	-	-	-	**(+)**	-	**+**	-	-	-	-	-	-	-	NEG.
19.	24	**(17.750)**	2.512	-	-	-	-	-	-	-	NEG.	-	-	-	-	-	-	**+**	-	-	-	-	-	-	-	NEG.
20.	25	**31.473**	1.576	-	-	-	-	-	-	-	NEG.	-	-	-	-	-	-	**+**	-	-	-	-	-	-	-	NEG.
21.	26	**(19.382)**	**164.502**	-	**+**	-	-	-	-	-	NEG.	**+**	**+**	**+**	-	**+**	-	**+**	-	-	-	-	-	-	-	POS.
22.	24	**(17.000)**	1.500	-	-	-	-	-	-	-	NEG.	-	-	-	-	-	-	**+**	-	-	**(+)**	-	-	-	-	NEG.
23.	28	**(16.006)**	**>200**	-	**+**	-	**(+)**	**(+)**	**+**	-	POS.	**+**	-	-	-	-	-	**+**	-	**+**	-	-	-	-	-	POS.
24.	24	1.073	**77.237**	-	**+**	-	**(+)**	**(+)**	-	-	BOR.	-	-	-	-	-	**+**	**+**	**+**	**+**	-	-	-	-	-	POS.
25.	26	3.389	11.900	-	-	-	-	-	-	-	NEG.	-	-	-	-	-	**+**	**+**	-	-	-	-	-	-	**+**	POS.
26.	27	5.636	**91.750**	-	-	-	-	-	-	-	NEG.	**(+)**	**(+)**	-	-	-	**+**	**+**	**+**	**+**	**(+)**	-	-	-	-	POS.
27.	21	**(17.666)**	**28.134**	-	-	-	-	-	-	-	NEG.	-	-	-	-	-	**(+)**	**+**	**(+)**	**+**	-	-	-	-	-	BOR.
28.	22	**(16.454)**	3.746	-	**(+)**	-	-	-	-	-	NEG.	-	-	-	-	-	-	-	-	-	-	-	-	-	-	NEG.
29.	26	**27.129**	**48.575**	-	-	**(+)**	-	-	-	-	NEG.	**+**	**+**	**+**	-	-	**+**	**+**	-	-	-	-	-	-	-	POS.
30.	25	**34.035**	5.906	-	-	-	-	-	-	-	NEG.	**+**	-	-	-	-	-	**+**	-	-	**+**	**(+)**	-	-	**(+)**	POS.
31.	27	**(21.782)**	1.802	-	**+**	-	-	-	**(+)**	-	BOR.	-	-	-	-	-	-	**+**	-	-	-	-	-	-	-	NEG.

32.	28	15.761	**30.248**	-	-	-	-	-	-	-	NEG.	-	-	-	-	-	+	(+)	-	-	-	-	-	-	-	BOR.
33.	24	**24.253**	**24.747**	-	(+)	+	+	+	(+)	-	POS.	(+)	-	+	-	-	-	+	+	+	-	-	-	-	-	POS.
34.	21	**54.937**	**(18.792)**	-	-	-	+	+	+	-	POS.	-	-	+	-	-	-	+	-	+	-	-	-	-	-	POS.
35.	25	**41.873**	**>200**	-	+	-	+	+	(+)	-	POS.	+	+	+	-	-	-	+	+	-	-	-	-	(+)	+	POS.
36.	21	7.400	12.592	-	-	-	-	-	-	-	NEG.	-	-	-	-	-	+	+	-	-	-	-	-	-	-	BOR.
37.	24	**27.506**	9.898	-	(+)	(+)	-	-	-	-	NEG.	-	-	-	-	-	-	+	+	+	-	-	-	-	-	POS.
38.	21	**(21.832)**	**41.979**	-	-	-	-	-	-	-	NEG.	+	(+)	+	-	-	-	+	(+)	-	-	-	-	-	-	POS.
39.	22	**25.003**	**(18.792)**	-	(+)	-	-	-	-	-	NEG.	-	-	-	-	-	-	+	-	+	-	-	-	-	-	BOR.
40.	26	11.188	**(19.764)**	-	(+)	-	(+)	-	-	-	BOR.	-	-	-	-	-	-	+	+	+	-	-	-	-	-	POS.
41.	25	**49.467**	**82.707**	-	-	-	+	+	(+)	-	POS.	+	-	+	-	-	-	+	-	-	-	-	-	-	-	POS.
42.	24	**134.142**	**163.413**	-	+	(+)	+	+	+	-	POS.	+	(+)	+	-	-	(+)	+	+	-	-	-	-	-	-	POS.
43.	28	**28.143**	6.765	-	-	-	-	-	-	-	NEG.	-	-	-	-	-	-	+	(+)	+	-	-	-	(+)	-	BOR.
44.	27	15.369	**27.095**	-	-	+	(+)	(+)	-	-	POS.	-	-	(+)	+	-	-	+	(+)	+	-	-	-	-	-	POS.
45.	28	**46.895**	8.958	-	-	-	+	(+)	-	-	POS.	-	-	-	-	-	-	+	-	-	-	-	-	-	-	NEG.
46.	24	6.091	**96.061**	-	(+)	-	-	-	-	-	NEG.	-	+	+	-	-	-	+	-	-	-	-	-	-	-	POS.
47.	25	11.982	**90.463**	-	-	-	-	-	-	-	NEG.	-	-	-	-	-	(+)	+	-	-	-	-	-	-	-	NEG.
48.	26	**56.684**	4.170	-	-	-	+	+	(+)	-	POS.	-	-	-	-	-	-	+	-	-	-	-	-	-	-	NEG.
49.	24	**>200**	**(16.903)**	-	+	-	+	+	+	-	POS.	-	-	-	-	-	-	+	-	+	-	-	-	-	-	BOR.
50.	28	**122.192**	**33.372**	-	(+)	-	-	-	+	-	POS.	-	-	-	-	-	-	+	(+)	(+)	-	-	-	-	-	NEG.
51.	24	13.531	7.811	-	(+)	-	(+)	-	-	-	BOR.	-	-	-	-	-	-	(+)	+	+	-	-	-	-	-	POS.
52.	26	**63.496**	**29.177**	-	(+)	-	+	+	+	-	POS.	-	-	+	-	-	-	-	-	+	-	-	-	-	-	POS.
53.	27	**76.295**	8.429	-	-	-	+	+	(+)	-	POS.	-	-	-	-	-	-	+	+	+	-	-	-	-	-	POS.
54.	21	**>200**	**68.664**	+	+	-	(+)	-	+	-	POS.	-	+	(+)	-	-	-	+	-	-	-	-	-	-	-	POS.
55.	22	**91.592**	4.455	-	-	-	(+)	-	+	-	POS.	-	-	-	-	-	(+)	+	(+)	-	-	-	-	-	-	NEG.
56.	26	**43.567**	6.972	-	(+)	-	+	+	+	-	POS.	-	(+)	-	-	-	-	+	-	-	-	-	-	-	-	BOR.
57.	25	**32.780**	**40.379**	-	(+)	-	(+)	-	(+)	-	BOR.	-	(+)	(+)	-	-	-	+	-	-	-	-	-	(+)	-	BOR.
58.	27	4.808	4.112	-	-	-	-	-	-	-	NEG.	-	-	-	-	-	-	+	-	-	-	-	-	-	-	NEG.
59.	28	**>200**	**73.670**	-	+	-	+	+	+	-	POS.	-	(+)	-	-	-	-	+	+	+	-	-	-	-	-	POS.
60.	24	**27.911**	11.454	-	-	-	+	-	(+)	-	POS.	-	(+)	-	-	-	-	+	-	-	-	-	-	-	-	BOR.
61.	21	**95.248**	4.860	-	+	-	+	+	+	-	POS.	-	-	-	-	-	-	+	-	-	-	-	-	-	-	NEG.
62.	25	1.446	2.729	-	-	-	-	-	-	-	NEG.	-	-	-	-	-	-	-	-	-	-	+	-	-	-	BOR.
63.	28	**54.097**	4.172	-	-	-	+	+	+	-	POS.	-	-	-	-	-	-	+	-	-	-	-	-	-	-	NEG.
64.	24	**30.663**	**161.198**	-	(+)	-	+	+	+	-	POS.	-	+	+	-	-	-	+	-	+	-	-	-	-	-	POS.
65.	21	**41.414**	8.699	-	-	-	-	-	-	-	NEG.	-	-	+	-	-	+	-	+	-	-	-	-	-	-	POS.
66.	22	**25.035**	**31.171**	-	(+)	-	-	-	-	-	NEG.	-	(+)	+	-	-	-	+	-	-	-	-	-	-	-	POS.
67.	26	**22.190**	**29.435**	-	-	-	-	-	-	-	NEG.	(+)	-	+	-	-	+	+	(+)	+	-	-	-	-	(+)	POS.
68.	25	11.779	**56.263**	-	(+)	-	(+)	-	-	-	BOR.	-	-	-	-	(+)	(+)	+	-	-	-	-	-	-	-	NEG.
69.	24	**28.954**	**33.757**	-	-	-	(+)	-	(+)	-	BOR.	-	-	+	-	-	-	-	-	+	-	-	-	-	-	POS.
70.	28	**18.590**	**123.244**	-	(+)	-	-	-	-	-	NEG.	+	+	+	-	(+)	+	+	-	-	-	(+)	-	-	(+)	POS.
71.	22	**17.587**	**37.982**	-	(+)	-	-	-	-	-	NEG.	-	(+)	-	-	-	-	-	-	-	-	-	-	-	-	BOR.
72.	22	**37.535**	**116.515**	-	-	-	-	-	-	-	NEG.	+	+	+	-	-	-	+	(+)	+	-	-	-	-	+	POS.

73.	25	**171.940**	**44.186**	-	(+)	-	+	+	+	-	POS.	+	+	+	-	-	-	+	+	+	-	-	-	-	-	POS.
74.	28	**136.380**	**83.901**	-	+	-	(+)	+	+	-	POS.	+	+	+	-	-	-	+	-	-	(+)	-	-	-	-	POS.
75.	28	**71.700**	6.110	-	-	-	-	-	+	-	BOR.	-	-	-	-	-	+	+	(+)	+	-	-	-	-	-	POS.
76.	27	**19.400**	**25.044**	-	-	-	-	(+)	-	-	NEG.	(+)	-	-	-	-	-	-	-	-	-	-	-	-	-	NEG.
77.	26	**198.190**	5.600	-	(+)	-	+	+	+	-	POS.	-	-	-	-	-	-	+	-	-	-	-	-	-	-	NEG.
78.	25	**190.870**	**21.005**	-	-	-	+	+	+	-	POS.	-	-	+	-	-	-	+	-	+	-	-	-	-	-	POS.
79.	24	10.260	3.217	-	-	-	-	-	-	-	NEG.	+	-	(+)	-	-	-	+	+	-	+	-	-	-	-	POS.
80.	28	**16.192**	**19.907**	-	-	-	-	-	-	-	NEG.	-	-	-	-	-	-	+	-	-	-	-	-	-	-	NEG.

Legend:

IB: „-" NEG - negative result; „+" POS - positive result; **„(+)"** BOR - borderline result

ELISA: positive/borderline test results marked in bold type ; „>200" - value higher than concentration of K3 calibrator (>200 RU/ml)

B.a - *Borrelia afzelii*

B.b - *Borrelia burgdorferi*

B.g - *Borrelia garinii*

B.sp - *Borrelia spielmanii*

Table S2. Detailed summary of enzyme-linked immunosorbent (ELISA) and immunoblot (IB) assays results for anti-*Borrelia* IgM and IgG antibodies in sera samples of healthy individuals (control group).

	**ELISA**		**IB - IgM**								**IB - IgG**														
**No. of patient**	**IgM**	**IgG**	VlsE B.b	Flagellin	BmpA	OspC B.a	OspC B.b	OspC B.g	OspC B.sp	**Result**	VlsE B.a	VlsE B.b	VlsE B.g	Lipid B.a	Lipid B.b	p83	Flagellin	BmpA	OspC	p58 B.b	p21 B.b	p20 B.b	p19 B.b	p18 B.b	**Result**
1.	7.906	1.535	-	-	-	-	-	-	-	NEG.	-	-	-	-	-	-	+	-	-	-	-	-	-	-	NEG.
2.	8.862	0.651	-	-	-	-	-	-	-	NEG.	-	-	-	-	-	-	-	-	-	-	-	-	-	-	NEG.
3.	15.006	1.628	-	(+)	-	-	-	-	-	NEG.	-	-	-	-	-	-	+	-	-	-	-	-	-	-	NEG.
4.	**82.754**	1.023	-	(+)	-	-	-	-	-	NEG.	-	-	-	-	-	-	-	-	-	-	-	-	-	-	NEG.
5.	5.206	**20.382**	-	-	-	-	-	-	-	NEG.	-	-	-	-	-	-	+	-	-	-	-	-	-	+	BOR.
6.	9.763	15.437	-	-	-	-	-	-	-	NEG.	-	-	-	-	-	-	+	-	-	-	-	-	-	-	NEG.
7.	3.631	5.892	-	-	-	-	-	-	-	NEG.	-	-	-	-	-	-	+	(+)	-	-	-	-	-	-	NEG.
8.	8.244	13.339	-	(+)	-	-	-	-	-	NEG.	-	-	-	-	-	-	+	-	-	-	-	-	-	-	NEG.
9.	10.944	10.687	-	-	-	-	-	-	-	NEG.	-	-	-	-	-	(+)	+	-	-	-	-	-	-	-	NEG.
10.	3.744	**33.736**	-	-	-	-	-	-	-	NEG.	-	-	-	-	-	-	-	-	-	-	-	-	-	-	NEG.
11.	4.813	4.571	-	-	-	-	-	-	-	NEG.	-	-	-	-	-	-	+	-	+	-	-	-	-	-	BOR.
12.	9.313	3.876	-	-	-	-	-	-	-	NEG.	-	-	-	-	-	-	-	-	-	-	-	-	-	-	NEG.
13.	1.484	6.378	-	(+)	-	-	-	-	-	NEG.	-	-	-	-	-	-	-	-	-	-	-	-	-	-	NEG.
14.	2.113	1.628	-	(+)	-	(+)	(+)	-	-	BOR.	-	-	-	-	-	-	+	-	-	-	-	-	-	-	NEG.
15.	6.669	1.256	-	-	-	-	-	-	-	NEG.	-	-	-	-	-	-	+	-	-	-	-	-	-	-	NEG.
16.	11.45	8.255	-	-	-	-	-	-	-	NEG.	-	-	-	-	-	-	-	-	-	-	-	-	-	-	NEG.
17.	**19.831**	1.442	-	-	-	-	-	-	-	NEG.	-	-	-	-	-	-	+	-	-	-	-	-	-	-	NEG.
18.	2.338	8.950	-	-	-	-	-	-	-	NEG.	-	-	-	-	-	-	+	-	-	-	-	-	-	-	NEG.
19.	5.881	7.699	-	-	-	-	-	-	-	NEG.	-	-	-	-	-	-	-	-	-	-	-	-	-	-	NEG.
20.	1.613	2.764	-	-	-	-	-	-	-	NEG.	-	-	-	-	-	-	+	-	-	-	-	-	-	-	NEG.
21.	4.250	5.197	-	-	-	-	-	-	-	NEG.	-	-	-	-	-	-	-	-	-	-	-	-	-	-	NEG.
22.	12.631	1.581	-	-	-	-	-	-	-	NEG.	-	-	-	-	-	-	+	-	-	-	-	-	-	-	NEG.

Legend:

IB: „-" NEG - negative result; „+" POS - positive result; „(+)" BOR - borderline result

ELISA: positive/borderline test results marked in bold type ; „>200" - value higher than concentration of K3 calibrator (>200 RU/ml)

B.a - *Borrelia afzelii*

B.b - *Borrelia burgdorferi*

B.g - *Borrelia garinii*

B.sp - *Borrelia spielmanii*
